# Heterogeneity of progression-free survival surrogacy by sex in randomized trials testing immunotherapy in non-small cell lung cancer

**DOI:** 10.1093/jncics/pkaf085

**Published:** 2025-08-20

**Authors:** Eleonora Pagan, Isabella Sala, Laura Pala, Fabrizio Natali, Federico Merlo, Chiara Oriecuia, Claudia Specchia, Tommaso De Pas, Chiara Catania, Emilia Cocorocchio, Daniele Laszlo, Giovanni Ceresoli, Marzia Locatelli, Priscilla Cascetta, Flaminia Facella, Benedetta Tinterri, Martina Pino, Jacopo Canzian, Giuseppe Giaccone, Vincenzo Bagnardi, Fabio Conforti

**Affiliations:** Department of Statistics and Quantitative Methods, University of Milano-Bicocca, Milan, Italy; Department of Statistics and Quantitative Methods, University of Milano-Bicocca, Milan, Italy; Department of Medicine and Surgery, University of Milano-Bicocca, Milan, Italy; Department of Medical Oncology, Humanitas Gavazzeni, Bergamo, Italy; Department of Biomedical Sciences, Humanitas University, Milan, Italy; Department of Statistics and Quantitative Methods, University of Milano-Bicocca, Milan, Italy; Department of Statistics and Quantitative Methods, University of Milano-Bicocca, Milan, Italy; Department of Molecular and Translational Medicine, Università di Brescia, Brescia, Italy; Department of Molecular and Translational Medicine, Università di Brescia, Brescia, Italy; Department of Medical Oncology, Humanitas Gavazzeni, Bergamo, Italy; Department of Medical Oncology, Humanitas Gavazzeni, Bergamo, Italy; Department of Medical Oncology, Humanitas Gavazzeni, Bergamo, Italy; Department of Medical Oncology, Humanitas Gavazzeni, Bergamo, Italy; Department of Medical Oncology, Humanitas Gavazzeni, Bergamo, Italy; Department of Medical Oncology, Humanitas Gavazzeni, Bergamo, Italy; Department of Medical Oncology, Humanitas Gavazzeni, Bergamo, Italy; Department of Medical Oncology, Humanitas Gavazzeni, Bergamo, Italy; Department of Medical Oncology, Humanitas Gavazzeni, Bergamo, Italy; Department of Medical Oncology, Humanitas Gavazzeni, Bergamo, Italy; Department of Medical Oncology, Humanitas Gavazzeni, Bergamo, Italy; Meyer Cancer Center, Weill Cornel Medicine, New York, NY, United States; Department of Statistics and Quantitative Methods, University of Milano-Bicocca, Milan, Italy; Department of Medical Oncology, Humanitas Gavazzeni, Bergamo, Italy; Department of Biomedical Sciences, Humanitas University, Milan, Italy

## Abstract

**Background:**

The surrogacy of progression-free survival (PFS) for overall survival (OS) at the trial-level in randomized clinical trials (RCTs) testing immune checkpoint inhibitors (ICIs) in patients with advanced non-small cell lung cancers (NSCLC) is influenced by several clinical-pathological factors. However, potential heterogeneity of PFS surrogacy according to patients’ sex has never been investigated.

**Methods:**

RCTs testing ICIs as monotherapy or combined with chemotherapy in patients with advanced NSCLC reporting hazard ratios (HRs) for PFS and OS according to patients’ sex were included. The main objective was to assess sex-based heterogeneity in the trial-level association between PFS (surrogate endpoint) and OS (reference endpoint), overall and in subgroups defined by treatment type (ICIs as monotherapy vs ICIs plus chemotherapy). We used the coefficient of determination (*R*^2^) to quantify surrogacy.

**Results:**

Twenty RCTs, for a total of 7528 male and 3008 female patients, were included. Overall, the association between OS-HR and PFS-HR was moderate: the *R*^2^ from a model adjusted by the type of treatment administered in the experimental arm was 0.69 (95% confidence interval [CI] = 0.34 to 0.88). Sex-disaggregated analysis showed heterogeneity in PFS surrogacy: the association was strong in male patients (adjusted *R*^2^ = 0.77; 95% CI = 0.56 to 0.89), but poor in female (adjusted *R*^2^ = 0.31, 95% CI = 0.03 to 0.63). Consistent results were obtained in subgroups analyses by treatment type, and in cross-validation analysis.

**Conclusions:**

In RCTs testing ICIs alone or combined with chemotherapy in patients with advanced NSCLC, PFS is a robust surrogate endpoint for OS in male patients but not in female.

## Introduction

Surrogate endpoints represent a valuable tool in clinical research, especially in the oncology field, as they address the need to expedite drug approvals, allowing patients to have access to effective therapies faster, more efficiently, and more affordably, than waiting for final overall survival (OS) results of randomized clinical trials (RCTs).^1^^,^^2^ However, a rigorous demonstration of the value of a surrogate endpoint is required, to avoid exposing patients to potential toxicities of ineffective drugs and to prevent the community from bearing an unjustified financial burden.^1^^,^^2^

For decades, progression-free survival (PFS) has served as a surrogate endpoint for OS in RCTs testing chemotherapy in patients with advanced solid tumors.^1^ However, the validity of PFS as a surrogate has been questioned, particularly in the specific context of RCTs evaluating immune checkpoint inhibitors (ICIs), given the distinct mechanisms of action of this novel drug class.^3^

Indeed, these agents function through mechanisms distinct from traditional anticancer therapies by activating or restoring the immune system’s capacity to recognize and attack tumors. This can lead to delayed treatment effects, durable responses, and phenomena such as pseudo-progression, where tumors initially appear to enlarge before subsequently regressing. These unique features may result in delayed separation of PFS curves and the emergence of plateaus in the Kaplan-Meier curves for the ICI treatment arm. Consequently, these dynamics challenge the proportional hazards assumption, which underpins PFS hazard ratio (HR) estimation.^3^

Recently, a US Food and Drug Administration (FDA)–sponsored meta-analysis based on individual patient data (IPD) from 13 RCTs testing ICIs alone or combined with chemotherapy in patients with advanced non-small cell lung cancer (NSCLC), showed a strong correlation at the trial-level between treatment effects on PFS and OS overall.^4^ However, the strength of such correlation decreased, ranging from weak to moderate, when surrogacy was assessed according to PD-L1 expression levels by tumors and the specific type of treatment administered. Notably, the influence of patients’ sex in this context has never been investigated.^4^ Based on evidence from our previous studies and others indicating significant differences in clinical benefit from ICIs between male and female patients with NSCLC, we speculated that the surrogacy of PFS for OS might also vary according to patients’ sex.^5^^,^^6^ To investigate this hypothesis, we conducted a meta-analysis of all RCTs testing ICIs as monotherapy or in combination with chemotherapy in patients with advanced NSCLC.

## Methods

### Search strategy, selection criteria, and data extraction

We followed recommendations of the Preferred Reporting Items for Systematic reviews and Meta-Analyses and the Reporting of Surrogate Endpoint Evaluation using Meta-analyses guidelines to perform this systematic review and meta-analysis.^7^^,^^8^ We searched PubMed, for phase 2 or 3 RCTs testing ICIs, published from the inception of the database to December 31, 2023. We also reviewed abstracts and presentations from all major conference proceedings, from January 2010 to December 2023.

Two investigators (E.P. and F.C.) independently searched the databases. The search terms were “CTLA-4,” “cytotoxic T-lymphocyte-associated protein 4,” “PD-1,” “programmed death receptor 1,” “PD-L1,” “immune checkpoint inhibitor*,” “ipilimumab,” “tremelimumab,” “nivolumab,” “pembrolizumab,” “durvalumab,” “atezolizumab,” “cemiplimab,” “spartalizumab,” “avelumab,” “toripalimab,” “dostarlimab,” “balstilimab,” “penpulimab,” “retifanlimab,” and “sintilimab.”

We included RCTs (1) assessing PD-(L)1 inhibitors either as monotherapy or in combination with chemotherapy, in patients with advanced NSCLC; (2) randomizing at least 100 patients; and (3) displaying the HR for OS and PFS by patients’ sex.

We excluded single-arm phase 1 and 2 trials (ie, nonrandomized trials), RCTs conducted in (neo)adjuvant setting, RCTs considering ICIs as control arm (either monotherapy or combined with other therapies), or RCTs enrolling patients with EGFR mutated and/or ALK translocated tumors.

Titles, abstracts, and full-text articles were reviewed independently by four authors (F.C., L.P., E.P., I.S.). Inconsistencies were discussed by all authors to reach consensus. Reference lists of articles included in the final selection were reviewed to identify additional relevant papers. When duplicate publications were identified, only the most recent and complete were included.

Based on a predefined form, we extracted data on the following variables: study name, first author and year of publication, study design and blinding, trial phase, primary endpoint(s), underlying malignancy, number of patients, median follow-up time, line of therapy, type of experimental, and control treatment, OS-HR and PFS-HR in the intention-to-treat (ITT) population and according to patients’ sex.

### Quality assessment of trials

To ascertain risk of bias, we assessed the methodological quality of each trial using the Cochrane Risk of bias tool (version 5.2.0).^9^ Responses in each domain (random sequence generation, allocation concealment, blinding of participants and personnel, blinding of outcome assessment, incomplete outcome data and selective outcome reporting) were assessed as having a “low,” “unclear,” or “high” risk of bias.

### Statistical analysis

The main objective of the analysis was to assess sex-based heterogeneity in the trial-level association between the surrogate endpoint (ie, PFS) and the reference endpoint (ie, OS).

The value of PFS as surrogate endpoint for OS was assessed using a meta-analytical approach, considering the ITT population of the trials as well as the subgroups defined by patients’ sex.

The data extracted from the included three-arm trials were treated as two separate comparisons, with the control arm being duplicated in both comparisons. For this reason, our unit of analysis was the comparison between pairs of treatment arms and not the trial. Each pairwise comparison was categorized according to the specific type of treatment administered in the experimental arm: (1) ICI alone or (2) ICI plus chemotherapy.

First, we combined PFS and OS effect sizes for each pairwise treatment comparison using a random effects meta-analysis. The heterogeneity across trials was assessed with the Cochrane Q test and the *I*^2^ statistic.

Then, to quantify the association between the effect of treatment on the reference endpoint (ie, OS) and the effect of treatment on the surrogate endpoint (ie, PFS), we used a correlation approach based on weighted linear regression model. The model was weighted by the number of subjects randomized in each pairwise treatment comparison. In the model, treatment effects were fitted on a log scale. The coefficient of determination (*R*^2^) was used to quantify the surrogacy value at trial-level of PFS. The 95% confidence interval (CI) for *R*^2^ was estimated by bootstrap analysis with 1000 samples. As commonly applied in surrogate endpoint literature,^8^  *R*^2^ values at or greater than 0.7 suggest *strong* correlation (suggesting surrogacy), values between 0.69 and 0.5 a *moderate* correlation, and values less than 0.5 a *weak* correlation.

The slope of the regression line was also reported as an alternative measure of surrogacy. For the treatment effects to be associated, we required that the slope significantly differed from 0.

Moreover, to adjust the *R*^2^ for the type of treatment administered in the experimental arm, we also fitted multivariable weighted linear models including the covariate as adjustment variable. For the analysis on all comparisons, overall and stratified by patients’ sex, we reported also the adjusted *R*^2^—that is, the square of the partial correlation coefficient obtained from the multivariable model.

A leave-one-out cross-validation was performed to validate results obtained in the main analysis. Each comparison was left out once, and the surrogate model was built with the other comparisons; this model was then reapplied to the left-out trial to predict the effect of treatment on the OS reference endpoint. The leave-one-out cross-validated *R*^2^ was calculated as the correlation between the individual predictions made by the model over all comparisons and the actual treatment effects.

Finally, we calculated the surrogate threshold effect (STE), defined as the minimum value of PFS-HR necessary to predict a statistically significant OS benefit in a future trial. Graphically, STE is the intersection of the upper limit of the 95% prediction band and the horizontal line representing the predicted OS-HR equal to 1 (null effect). The 95% prediction band was calculated from the weighted regression model used to derive the coefficient of determination *R*^2^ and was based on the predicted weight assigned to the HR for a future trial. In the calculation of the prediction band, we considered a future trial with an expected sample size equal to the average number of patients enrolled in the included comparisons.

Analyses were performed with SAS software v9.4 (SAS Institute, Cary, North Carolina, USA) and R software v3.6.0.

## Results

Overall, 20 RCTs for a total of 7528 male and 3008 female patients were included in the analysis (Figure S1).^10-38^

Table 1 shows the main features of included trials. One phase 2/3 trial and 19 phase 3 trials were included. Fifteen studies were in the first-line setting and 5 in further lines of treatment. Ten trials enrolled patients with both squamous and nonsquamous NSCLC, 4 with only squamous and 6 with only nonsquamous tumors. OS alone was the primary endpoint in 6 trials, PFS alone in 6 trials, whereas OS and PFS were coprimary endpoints in 8 trials.

**Table 1.** Overview of included trials.

Trial	Publication	Phase	Line	Histotype	Primary endpoint(s)	Experimental arm	Control arm	Type of treatment (experimental arm)	Subgroup	*N*	OS-HR (95% CI)	PFS-HR (95% CI)
CameL	Zhou C et al. (2023)^10^	3	I	Nonsquamous	PFS	Camrelizumab + Pemetrexed + Carboplatin	Pemetrexed + Carboplatin	ICI + CT	Female	117	0.57 (0.35 to 0.92)	0.62 (0.41 to 0.95)
									Male	295	0.78 (0.59 to 1.03)	0.53 (0.41 to 0.68)
									Overall	412	0.72 (0.57 to 0.92)	0.55 (0.44 to 0.69)
CameL-sq	Ren S et al. (2022)^11^	3	I	Squamous	PFS	Camrelizumab + Chemotherapy	Carboplatin + Paclitaxel	ICI + CT	Female	30	1.06 (0.21 to 5.26)	0.97 (0.40 to 2.34)
									Male	359	0.55 (0.40 to 0.75)	0.35 (0.27 to 0.46)
									Overall	389	0.55 (0.40 to 0.75)	0.37 (0.29 to 0.47)
CheckMate 017	Brahmer J et al. (2015)^12^ and Borghaei H et al. (2021)^14^	3	>I	Squamous	OS	Nivolumab	Docetaxel	ICI alone	Female	64	0.67 (0.36 to 1.25)	0.71 (0.40 to 1.26)
									Male	208	0.57 (0.41 to 0.78)	0.63 (0.46 to 0.85)
									Overall	272	0.62 (0.48 to 0.79)	0.61 (0.47 to 0.80)
CheckMate 026	Carbone DP et al. (2017)^13^	3	I	Both	PFS	Nivolumab	Investigator’s Choice of Chemotherapy	ICI alone	Female	162	1.00 (0.66 to 1.53)	1.20 (0.82 to 1.74)
									Male	261	0.98 (0.72 to 1.33)	1.14 (0.84 to 1.53)
									Overall	423	1.02 (0.80 to 1.30)	1.15 (0.91 to 1.45)
CheckMate 057	Borghaei H et al. (2015)^37^ and Borghaei H et al. (2021)^14^	3	>I	Nonsquamous	OS	Nivolumab	Docetaxel	ICI alone	Female	263	0.78 (0.58 to 1.04)	1.04 (0.80 to 1.37)
									Male	319	0.73 (0.56 to 0.96)	0.81 (0.63 to 1.04)
									Overall	582	0.70 (0.58 to 0.83)	0.90 (0.75 to 1.08)
CheckMate 078	Chang J et al. (2022)^15^ and Lu S et al. (2021)^38^	3	>I	Both	OS	Nivolumab	Docetaxel	ICI alone	Female	107	0.78 (0.50 to 1.20)	1.08 (0.69 to 1.73)
									Male	397	0.77 (0.61 to 0.96)	0.72 (0.57 to 0.89)
									Overall	504	0.75 (0.61 to 0.93)	0.78 (0.64 to 0.96)
CHOICE-01	Wang Z et al. (2023)^16^	3	I	Both	PFS	Toripalimab + Chemotherapy	Placebo + Chemotherapy	ICI + CT	Female	88	0.61 (0.30 to 1.30)	0.58 (0.35 to 1.00)
									Male	377	0.73 (0.54 to 0.98)	0.50 (0.39 to 0.64)
									Overall	465	0.69 (0.53 to 0.92)	0.49 (0.39 to 0.61)
EMPOWER-Lung 1	Özgüroğlu M et al. (2023)^17^	3	I	Both	OS + PFS	Cemiplimab	Investigator’s Choice of Platinum-based Chemotherapy	ICI alone	Female	85	0.78 (0.43 to 1.44)	0.83 (0.49 to 1.41)
									Male	480	0.53 (0.42 to 0.67)	0.46 (0.37 to 0.57)
									Overall	565	0.57 (0.46 to 0.71)	0.51 (0.41 to 0.62)
EMPOWER-Lung 3	Makharadze T et al. (2023)^18^	3	I	Both	OS	Cemiplimab + Chemotherapy	Placebo + Chemotherapy	ICI + CT	Female	75	0.98 (0.54 to 1.78)	0.71 (0.42 to 1.20)
									Male	391	0.55 (0.42 to 0.71)	0.48 (0.38 to 0.62)
									Overall	466	0.65 (0.51 to 0.82)	0.55 (0.44 to 0.68)
IMpower130	West H et al. (2019)^19^	3	I	Nonsquamous	OS + PFS	Atezolizumab + Carboplatin + Nab-Paclitaxel	Carboplatin + Nab-Paclitaxel	ICI + CT	Female	279	0.66 (0.46 to 0.93)	0.59 (0.45 to 0.78)
									Male	400	0.87 (0.66 to 1.15)	0.67 (0.54 to 0.85)
									Overall	679	0.79 (0.64 to 0.98)	0.64 (0.54 to 0.77)
IMpower131	Jotte R et al. (2020)^20^	3	I	Squamous	OS + PFS	Atezolizumab + Carboplatin + Nab-Paclitaxel	Carboplatin + Nab-Paclitaxel	ICI + CT	Female	126	0.68 (0.44 to 1.04)	0.66 (0.45 to 0.97)
									Male	557	0.91 (0.75 to 1.12)	0.71 (0.59 to 0.85)
									Overall	683	0.88 (0.73 to 1.05)	0.71 (0.60 to 0.85)
IMpower132	Nishio M et al. (2021)^21^	3	I	Nonsquamous	OS + PFS	Atezolizumab + Pemetrexed + Cisplatin/Carboplatin	Pemetrexed + Cisplatin/Carboplatin	ICI + CT	Female	194	0.76 (0.54 to 1.09)	0.51 (0.36 to 0.71)
									Male	384	0.93 (0.73 to 1.18)	0.64 (0.51 to 0.79)
									Overall	578	0.86 (0.71 to 1.06)	0.60 (0.49 to 0.72)
IPSOS	Lee SM et al. (2023)^22^	3	I	Both	OS	Atezolizumab	Single-agent chemotherapy	ICI alone	Female	125	0.86 (0.58 to 1.27)	1.04 (0.70 to 1.52)
									Male	328	0.76 (0.59 to 0.98)	0.76 (0.59 to 0.97)
									Overall	453	0.78 (0.63 to 0.97)	0.87 (0.70 to 1.07)
JAVELIN Lung 100	Reck M et al.(2024)	3	I	Both	OS + PFS	Avelumab 10 mg/kg every 2 weeks	Platinum-based doublet chemotherapy	ICI alone	Female	97	1.10 (0.69 to 1.75)	0.94 (0.56 to 1.58)
									Male	270	0.77 (0.58 to 1.03)	0.66 (0.48 to 0.90)
									Overall	367	0.85 (0.67 to 1.09)	0.71 (0.54 to 0.93)
JAVELIN Lung 100	Reck M et al. (2024)^23^	3	I	Both	OS + PFS	Avelumab 10 mg/kg once weekly for 12 weeks, followed by avelumab 10 mg/kg every 2 weeks thereafter	Platinum-based doublet chemotherapy	ICI alone	Female	66	0.85 (0.47 to 1.54)	0.89 (0.48 to 1.65)
									Male	193	0.78 (0.55 to 1.10)	0.67 (0.46 to 0.97)
									Overall	259	0.79 (0.59 to 1.07)	0.72 (0.52 to 0.98)
JAVELIN Lung 200	Park K et al. (2021)^24^ and Barlesi F et al. (2018)^30^	3	>I	Both	OS	Avelumab	Docetaxel	ICI alone	Female	162	1.02 (0.72 to 1.45)	1.93 (1.24 to 3.01)
									Male	367	0.82 (0.65 to 1.03)	0.77 (0.59 to 1.01)
									Overall	529	0.87 (0.71 to 1.05)	1.01 (0.80 to 1.28)
KEYNOTE-010	Herbst RS et al. (2021)^25^ and Herbst RS et al. (2016)^31^	2/3	>I	Both	OS + PFS	Pembrolizumab	Docetaxel	ICI alone	Female	399	0.66 (0.53 to 0.84)	1.02 (0.78 to 1.32)
									Male	634	0.71 (0.60 to 0.86)	0.78 (0.64 to 0.94)
									Overall	1033	0.70 (0.61 to 0.80)	0.84 (0.73 to 0.96)
KEYNOTE-024	Reck M et al. (2019)^32^ and Reck M et al. (2016)^33^ and Reck M et al. (2021)^26^	3	I	Both	PFS	Pembrolizumab	Investigator’s Choice of Platinum-based Chemotherapy	ICI alone	Female	118	0.95 (0.56 to 1.62)	0.75 (0.46 to 1.21)
									Male	187	0.54 (0.36 to 0.79)	0.39 (0.26 to 0.58)
									Overall	305	0.62 (0.48 to 0.81)	0.50 (0.39 to 0.65)
KEYNOTE-189	Rodríguez-Abreu D et al. (2021)^34^ and Garassino MC et al. (2023)^23^	3	I	Nonsquamous	OS + PFS	Pembrolizumab + Cisplatin/Carboplatin + Pemetrexed	Placebo + Cisplatin/Carboplatin + Pemetrexed	ICI + CT	Female	253	0.41 (0.30 to 0.56)	0.39 (0.29 to 0.52)
									Male	363	0.74 (0.56 to 0.96)	0.58 (0.46 to 0.74)
									Overall	616	0.60 (0.50 to 0.72)	0.50 (0.42 to 0.60)
KEYNOTE-407	Paz-Ares L et al. (2018)^35^ and Novello S et al. (2023)^28^	3	I	Squamous	OS + PFS	Pembrolizumab + Carboplatin-Paclitaxel/Nab-Paclitaxel	Placebo + Carboplatin-Paclitaxel/Nab-Paclitaxel	ICI + CT	Female	104	0.42 (0.22 to 0.81)	0.49 (0.30 to 0.81)
									Male	455	0.69 (0.51 to 0.94)	0.58 (0.46 to 0.73)
									Overall	559	0.71 (0.59 to 0.85)	0.62 (0.52 to 0.74)
ORIENT-11	Zhang L et al. (2022)^29^ and Yang Y et al. (2020)^36^	3	I	Nonsquamous	PFS	Sintilimab + Pemetrexed + Cisplatin/Carboplatin	Pemetrexed + Cisplatin/Carboplatin	ICI + CT	Female	94	0.99 (0.56 to 1.77)	0.60 (0.33 to 1.10)
									Male	303	0.57 (0.43 to 0.77)	0.44 (0.32 to 0.61)
									Overall	397	0.65 (0.50 to 0.85)	0.48 (0.36 to 0.64)

Abbreviations: CI = confidence interval; CT = chemotherapy; HR = hazard ratio; ICI = immune checkpoint inhibitor; OS = overall survival; PFS = progression-free survival.

The publication years spanned from 2015 to 2023, with a median follow-up of 35 months (min-max range = 7-70). The sample size of trials ranged from 259 to 1033 patients.

One trial had three treatment arms, for a total of 21 comparisons analyzed. Eleven comparisons tested ICI alone, whereas 10 comparisons evaluated the combination of ICI with chemotherapy.

Table S1 reports the quality assessment of trials according to the Cochrane Risk of bias tool. Overall, the quality of trials was high, as the risks of selection, attrition, reporting and other forms of bias for all the RCTs included in the analysis were low. The only potential biases affecting trials were performance and detection bias, as only 6 of 20 RCTs had a double-blind design.

Overall, the OS-HR ranged between 0.55 and 1.02, and the PFS-HR between 0.37 and 1.15.

**Figure 1.**
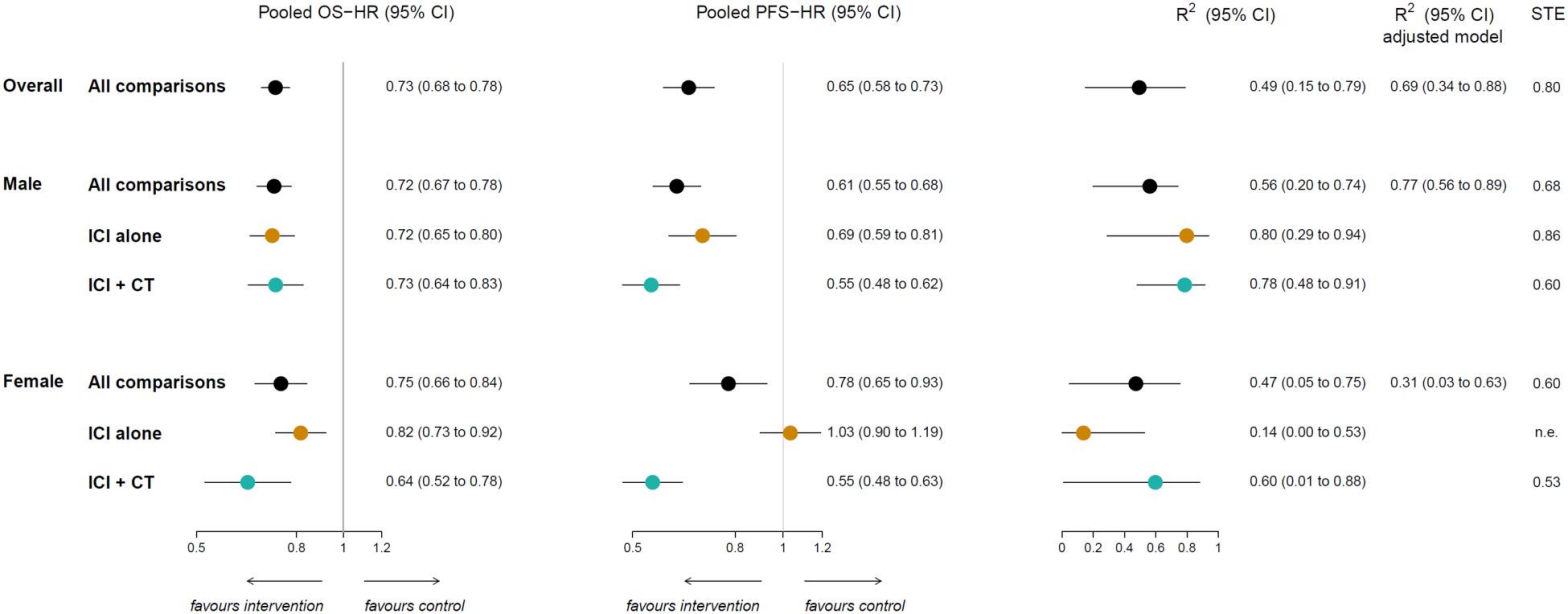
Forest plots showing meta-analytic pooled estimate (with 95% CI) of OS-HR and PFS-HR, *R*^2^ coefficient (with 95% CI) from the weighted linear regression models and STE, overall and according to sex and type of treatment administered in the experimental arm. The figure shows in the first and second panel the meta-analytic pooled estimate (circles) of treatment effects on OS-HR (first panel) and PFS-HR (second panel). Horizontal lines indicate the 95% CI and the solid vertical line indicates an HR of 1, which is the null-hypothesis value. Values less than 1 indicate a treatment effect in favor of the experimental arm, whereas values greater than 1 indicate treatment effects in favor of the control. The meta-analytic pooled estimates of OS-HR and PFS-HR are displayed overall and in subgroups according to patients’ sex, stratified by type of treatment administered in the experimental arm. The third and fourth panel show the unadjusted and adjusted *R*^2^ coefficients (with 95% CI) estimated from the weighted linear regression model. The last column reports the STE values, displayed overall and in subgroups according to patients’ sex, stratified by type of treatment administered in the experimental arm. Abbreviations: CI = confidence interval; CT = chemotherapy; HR = hazard ratio; ICI = immune checkpoint inhibitor; n.e. = not estimable; OS = overall survival; PFS = progression-free survival; STE = surrogate threshold effect.

We assessed the association between treatment effects on the final endpoint (ie, OS) vs treatment effects on the surrogate endpoint (ie, PFS) and estimated a regression equation based on data from all comparisons. In the ITT population, there was a moderate association between OS-HR and PFS-HR: the *R*^2^ was 0.49 (95% CI = 0.15 to 0.79), and the slope of the regression line was 0.40 (Figure 1 and Figure S2). After adjusting the model by the type of treatment administered in the experimental arm the *R*^2^ increased to 0.69 (95% CI = 0.34 to 0.88; Figure 1).

Sex-disaggregated analysis showed heterogeneity of PFS surrogacy according to patients’ sex. The association between OS-HR and PFS-HR was moderate in male patients, with an *R*^2^ of 0.56 (95% CI = 0.20 to 0.74; slope = 0.53; Figure 1 and Figure S2). The association became strong after the adjustment for type of treatment administered in the experimental arm (adjusted *R*^2^ = 0.77, 95% CI = 0.56 to 0.89; Figure 1). On the contrary, the association was poor in female patients, both in the unadjusted and adjusted model: the unadjusted *R*^2^ was 0.47 (95% CI = 0.05 to 0.75; slope = 0.47) and the adjusted *R*^2^ was 0.31 (95% CI = 0.03 to 0.63; Figure 1 and Figure S2).

The leave-one-out cross-validation analysis resulted in a cross-validated *R*^2^ equal to 0.71 for male patients and 0.26 for female (Figure S3).

The heterogeneity of PFS surrogacy according to patients’ sex was then assessed in subgroups defined by specific treatment type administered in the experimental arm.

**Figure 2.**
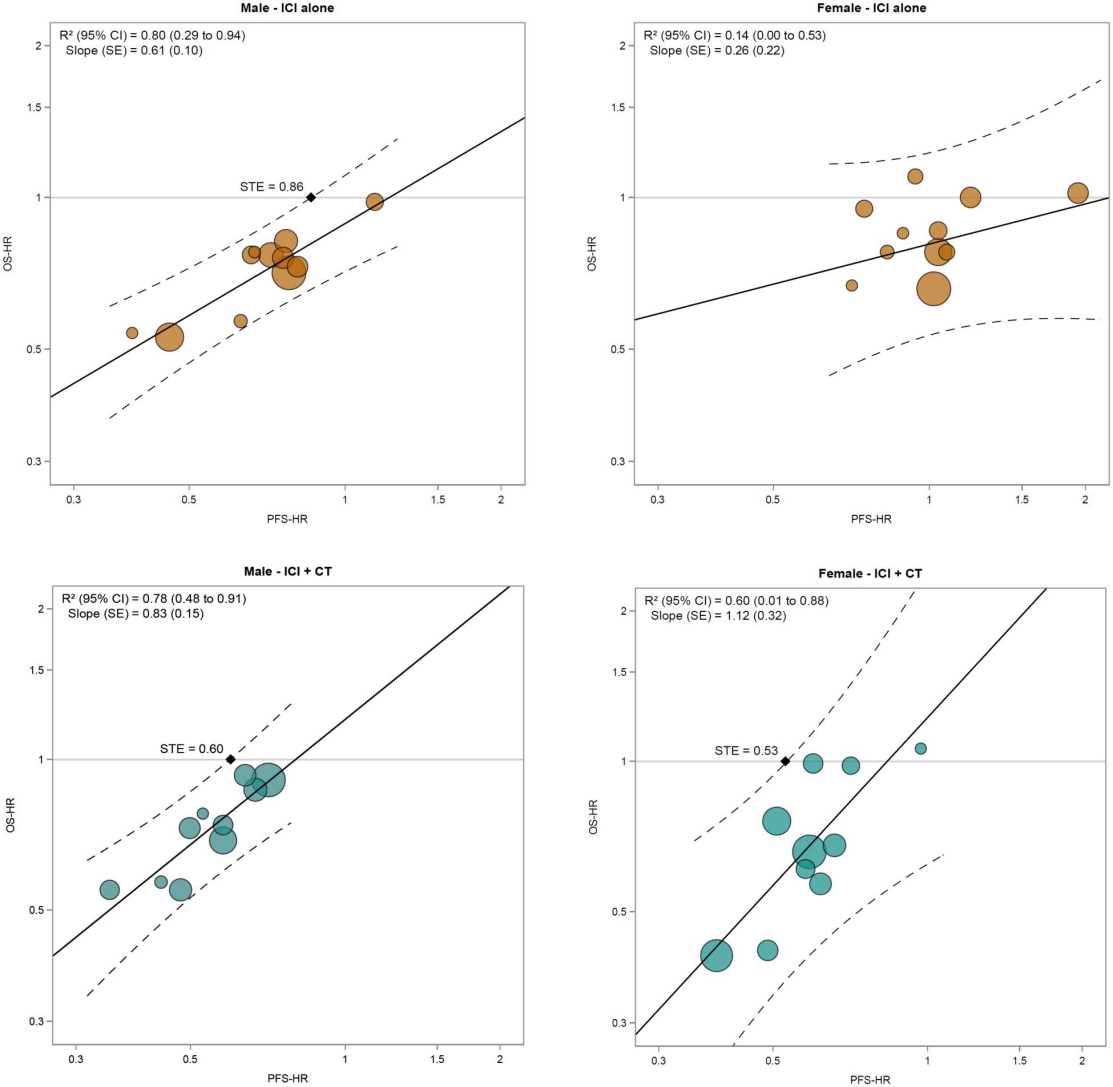
Correlation between treatment effects on OS-HR and PFS-HR, in subgroups defined by patients’ sex and type of treatment administered in the experimental arm. The figure shows the correlations between treatment effects on OS-HR and PFS-HR, in subgroups defined by patients’ sex (males in left panels and females in right panels) and treatment type (ICI alone in upper panels or ICIs plus chemotherapy in lower panels). Each circle represents a comparison, and the surface area of the circle is proportional to the number of patients in the corresponding comparison. Solid lines represent weighted regression lines and dotted lines the 95% prediction bands. Black diamonds represent the STE values. The *R*^2^ coefficients, with their 95% CI (square brackets), were reported in the legend. Abbreviations: CI = confidence interval; CT = chemotherapy; HR = hazard ratio; ICI = immune checkpoint inhibitor; OS = overall survival; PFS = progression-free survival; SE = standard error; STE = surrogate threshold effect.

In trials testing ICIs as monotherapy, the association between OS-HR and PFS-HR was strong in male patients: the *R*^2^ was 0.80 (95% CI = 0.29 to 0.94), and the slope of the regression line was 0.61 (Figures 1 and 2). On the contrary, the association was poor in female: the *R*^2^ was 0.14 (95% CI 0.00 to 0.53), and the slope of the regression line was 0.26 (Figures 1 and 2). The leave-one-out cross-validation analysis yielded a cross-validated *R*^2^ of 0.72 for male patients and 0.24 for female (Figure S3).

In trials testing ICIs plus chemotherapy, the association between OS-HR and PFS-HR was strong in male patients: the *R*^2^ was 0.78 (95% CI = 0.48 to 0.91), and the slope of the regression line was 0.83 (Figures 1 and 2). On the contrary, the association was moderate in female patients, with an *R*^2^ of 0.60 (95% CI = 0.01 to 0.88), and a slope of the regression line equal to 1.12 (Figures 1 and 2). The leave-one-out cross-validation analysis resulted in cross-validated *R*^2^ of 0.67 for male patients and 0.44 for female (Figure S3).

Finally, in male patients the STE was 0.68 overall, and 0.86 and 0.60 in RCTs testing ICIs as monotherapy or combined with chemotherapy, respectively (Figure 1). In female patients, the STE was 0.60 overall, not estimable in trials testing ICIs as monotherapy—as a consequence of the weak association observed between PFS and OS—and 0.53 in trials testing ICIs plus chemotherapy (Figure 1).

## Discussion

Progression-free survival has been long used as a surrogate endpoint for OS in RCTs testing chemotherapy and targeted therapy in patients with advanced solid tumors. However, its validity in trials testing ICIs has been questioned due to the unique mechanism of action of these drugs that relies on rehabilitating self-immunity against tumors, which could result in delayed clinical effects and long-term responders, as well as in initial disease progression followed by tumor shrinkage (ie, pseudo-progression events). In these instances, the PFS curves may take some time to separate, and the immunotherapy agent curve may have a long tail, leading to the violation of the proportional hazards assumption on which the calculation of HR is based.^39^

Our meta-analysis, which comprehensively reviewed all RCTs testing ICIs alone or with chemotherapy in advanced NSCLC, found a moderate trial-level correlation between treatment effects on PFS and OS in the ITT population. Notably, our findings align with and substantially extend those of a recent FDA-sponsored IPD meta-analysis by examining, for the first time, the impact of patient sex on the PFS-OS relationship at the trial-level. We observed significant heterogeneity by sex: there was a strong PFS-OS association in male patients but not in female patients.

In female patients, the *R*^2^ for PFS-OS association was 0.14 for ICI monotherapy and 0.60 for ICI-chemotherapy combinations, both below the 0.70 threshold recommended by international guidelines to indicate strong surrogacy.^8^ Conversely, in male patients, *R*^2^ exceeded 0.70 in both treatment settings. Moreover, STE analysis showed that a PFS-HR of about 0.7 overall, and as high as 0.86 for ICI monotherapy, was sufficient to predict a non-null OS-HR effect in male patients. Collectively, these findings support PFS as a reliable surrogate for OS in male patients.

As stated above, the results of the FDA-sponsored meta-analysis revealed that the surrogacy of PFS was influenced by the specific treatment type (ie, ICIs as monotherapy or in combination with chemotherapy) and PD-L1 expression levels by tumors.^4^ Although our findings demonstrated that the sex-based heterogeneity of PFS surrogacy holds across different treatment types, the unavailability of IPD precluded extending the same conclusions to PD-L1 tumor levels. Nevertheless, the independence of the effects of patients’ sex and PD-L1 levels on PFS surrogacy appears substantiated by the lack of a sex-based dimorphism in PD-L1 expression by NSCLC.^40^

The sex-based heterogeneity of PFS surrogacy highlights the need for more nuanced methodological approaches to evaluate new treatments efficacy and predicting patients’ clinical outcomes. The implications of our findings extend beyond methodological considerations to drug development strategies and regulatory decision-making processes, to ultimately ensure equitable access to effective therapies for all patients. Ideally, parallel studies should be conducted to independently evaluate new therapeutic strategies in male and female patient populations. However, given the practical challenges of this approach, regulatory agencies should, at a minimum, ensure that a standardized set of criteria is consistently applied to assess the impact of patient sex on both the efficacy and toxicity of emerging therapies. This includes incorporating sex as a stratifying factor in the design of future clinical trials testing immunotherapy, enrolling sufficiently large cohorts of both female and male patients, and planning sex-disaggregated analyses of results.

Furthermore, our findings also raise important questions regarding the identification of the potential biological factors driving the sex-based differences in PFS surrogacy. We and other groups showed that male and female patients with advanced NSCLC derived significantly different clinical benefits when treated with ICIs.^5^^,^^6^^,^^41-43^ Emerging evidence suggests that these sex-based disparities may arise from a complex interplay of genetic factors, sex hormones, microbiome composition, and behaviors.^44^^,^^45^

Among various factors, sex hormones appear to most strongly influence both ICI efficacy and resistance mechanisms. NSCLC tumor cells can locally produce high levels of estradiol via aromatase expression in the tumor microenvironment. Estradiol then induces PD-L1 expression through a paracrine mechanism, creating an immunosuppressive tumor microenvironment that limits ICI effectiveness. Conversely, androgens promote T cell exhaustion within tumors, facilitating immune evasion. These sex-specific immune responses may affect the ability of PFS to reliably reflect treatment effects on OS. Additionally, the higher incidence of adverse events, and subsequent treatment discontinuation, reported in female patients may further contribute to these differences.^44-47^

The main limitation of our analysis is that it is based on aggregated data rather than IPD. In the context of studies evaluating the surrogacy of PFS for OS at the trial-level, IPD enables the application of the hierarchical 2-stage model introduced by Burzykowski et al.^48^ Nevertheless, as reported in a simulation study coauthored by some of the same proponents of the two-stage approach based on IPD, the estimation performance of conventional measures using weighted least square regression on aggregate data is similar to that of the hierarchical two-stage model based on IPD.^49^ Therefore, an analysis based on IPD is unlikely to substantially change our conclusions.

Another possible limitation concerns the greater uncertainty in the estimates within the female subgroup. Because women are systematically underrepresented in the included RTCs, their estimates of OS-HR and PFS-HR tend to be less precise. Although uncertainty in the response variable (OS-HR) can introduce noise, it is the greater uncertainty in the explanatory variable (PFS-HR) that may lead to an attenuation of the observed association between the two endpoints. This may partially explain the apparently weaker surrogacy observed in the female subgroup.

Another limitation is that most included trials (14 of 20) were open-label, potentially introducing performance bias. This is particularly relevant for PFS, which relies on radiological assessment and may be influenced by knowledge of treatment allocation, possibly inflating treatment effects and affecting the PFS-OS correlation. However, all studies used blinded independent reviewers for PFS evaluation, reducing ascertainment bias. Still, performance bias may persist in other areas (eg, imaging frequency, supportive care) and cannot be fully ruled out in open-label designs.

Furthermore, it could be possible that the shape of the curves for PFS and thus its surrogacy value may be affected by other clinical and pathological factors that in turn could be associated with sex. However, it should be noted that our results are based on data from RCTs. The issue of confounding in subgroup analyses from RCTs is subtle. As reported by VanderWeele and Knol, “the effect of treatment within subgroups will not be confounded because treatment is randomized; but the effect of the secondary factor defining subgroups might be confounded since it is not randomized.”^50^ This means that we can safely conclude that the surrogacy value of PFS is different between males and females patients. On the contrary, we cannot exclude that the observed difference is not due to a causal effect of sex per se, rather than to other factors associated with sex.

An IPD meta-analysis of a large number of RCTs to better address this issue would therefore complement our findings, and we hope that the FDA may support such an analysis in the future.

In conclusion, our findings add to the debate on surrogate endpoints in oncology and underscore the need to consider sex-specific effects. For the first time, we show that in RCTs testing ICIs for advanced NSCLC, PFS is a reliable surrogate for OS in male but not in female patients. This nuanced understanding offers valuable insights for drug development and regulatory decisions. Going forward, validating surrogate endpoints and integrating sex-based analyses will be essential to advance precision oncology and personalized care in NSCLC and other cancers.

## Supplementary Material

pkaf085_Supplementary_Data

## Data Availability

Detailed extracted data on all included studies are available upon reasonable request to the corresponding author.
