# Supplementary material for: Heterogeneity of progression-free survival surrogacy by sex in randomized trials testing immunotherapy in non-small cell lung cancer
Source: JNCI Cancer Spectr. 2025 Aug 20;9(5):pkaf085. doi: 10.1093/jncics/pkaf085 (PMC12448438; doi:10.1093/jncics/pkaf085)
Supplement: pkaf085_Supplementary_Data [file pkaf085_supplementary_data.pdf]

SUPPLEMENTARY MATERIAL

Table S1. Quality assessment of trials according to the Cochrane Risk of bias tool........................2
Figure S1. PRISMA flow diagram. 3
Figure S2. Correlation between treatment effects on OS-HR and PFS-HR, overall and in subgroups according to patients' sex, stratified by type of treatment administered in the experimental arm. 4
Figure S3. Variation in R^2 value with leave-one-out cross validation. 5

Table S1. Quality assessment of trials according to the Cochrane Risk of bias tool.

Trial	Random sequence generation (selection bias)	Allocation concealment (selection bias)	Blinding of study participants and personnel (performance bias)	Blinding of outcome assessors (detection bias)	Incomplete outcome data (attrition bias)	Selective outcome reporting (reporting bias)	Other bias
CameL	Low	Low	High	Low	Low	Low	Low
CameL-sq	Low	Low	Low	Low	Low	Low	Low
CheckMate 017	Low	Low	High	Low	Low	Low	Low
CheckMate 026	Low	Low	High	Low	Low	Low	Low
CheckMate 057	Low	Low	High	Low	Low	Low	Low
CheckMate 078	Low	Low	High	Low	Low	Low	Low
CHOICE-01	Low	Low	Low	Low	Low	Low	Low
EMPOWER-Lung 1	Low	Low	High	Low	Low	Low	Low
EMPOWER-Lung 3	Low	Low	Low	Low	Low	Low	Low
IMpower130	Low	Low	High	Low	Low	Low	Low
IMpower131	Low	Low	High	Low	Low	Low	Low
IMpower132	Low	Low	High	Low	Low	Low	Low
IPSOS	Low	Low	High	Low	Low	Low	Low
JAVELIN Lung 100	Low	Low	High	Low	Low	Low	Low
JAVELIN Lung 200	Low	Low	High	Low	Low	Low	Low
KEYNOTE-010	Low	Low	High	Low	Low	Low	Low
KEYNOTE-024	Low	Low	High	Low	Low	Low	Low
KEYNOTE-189	Low	Low	Low	Low	Low	Low	Low
KEYNOTE-407	Low	Low	Low	Low	Low	Low	Low
ORIENT-11	Low	Low	Low	Low	Low	Low	Low

Figure S1. PRISMA flow diagram.

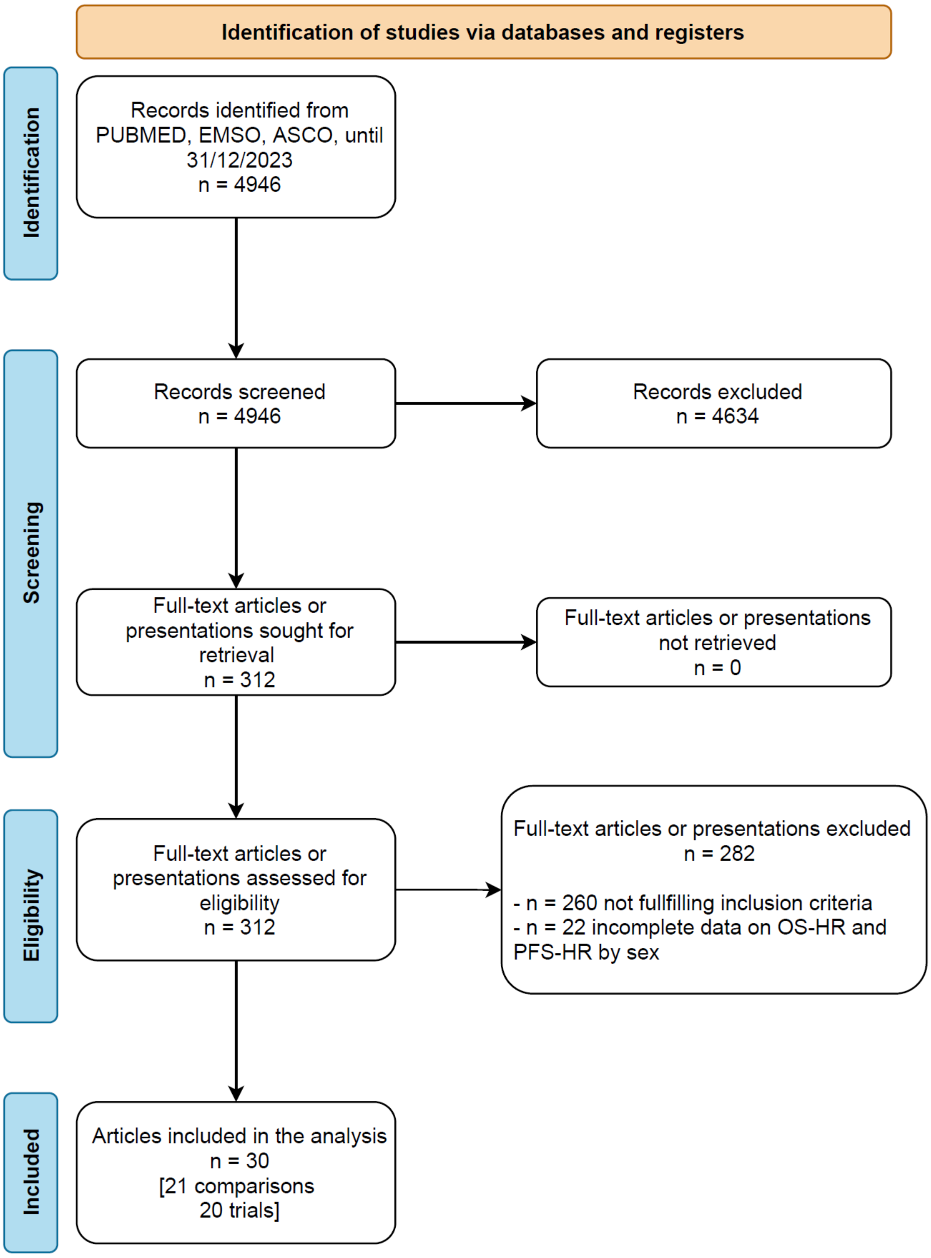

Figure S2. Correlation between treatment effects on OS-HR and PFS-HR, overall and in subgroups according to patients' sex, stratified by type of treatment administered in the experimental arm.

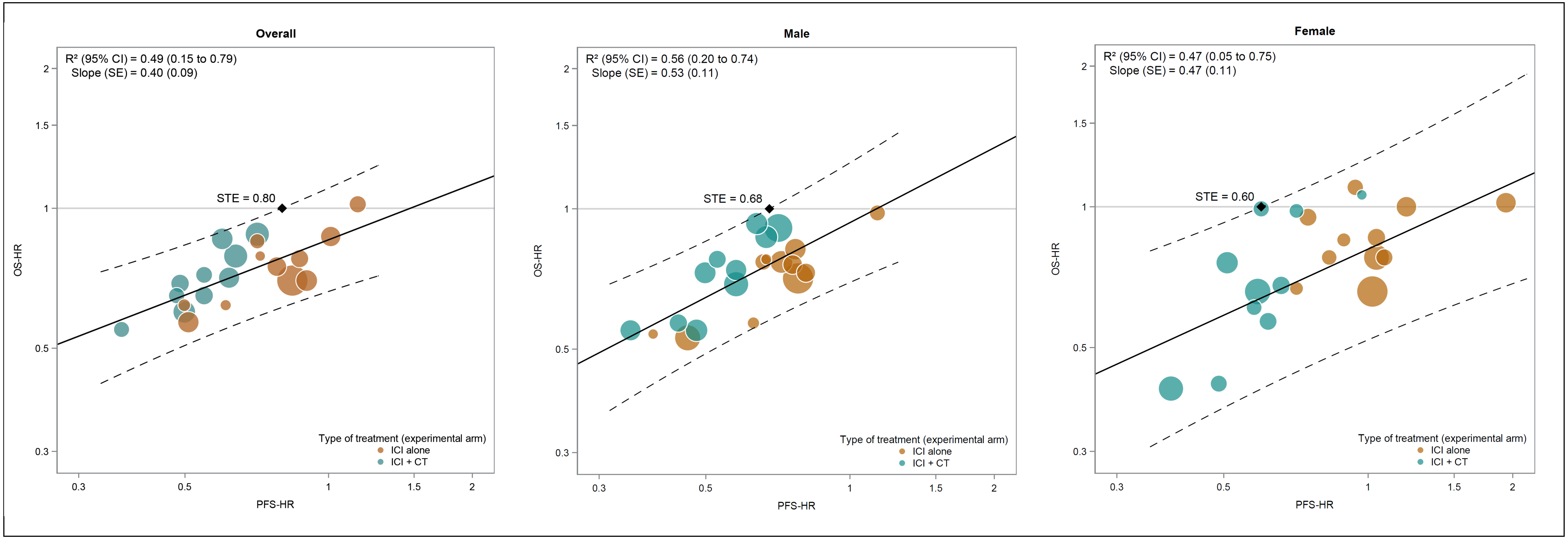

Abbreviations: CI, confidence interval; CT, chemotherapy; ICI, immune checkpoint inhibitor; HR, hazard ratio; OS, overall survival; PFS, progression-free survival; SE, standard error; STE, surrogate threshold effect.

Figure S3. Variation in R^2 value with leave-one-out cross validation.

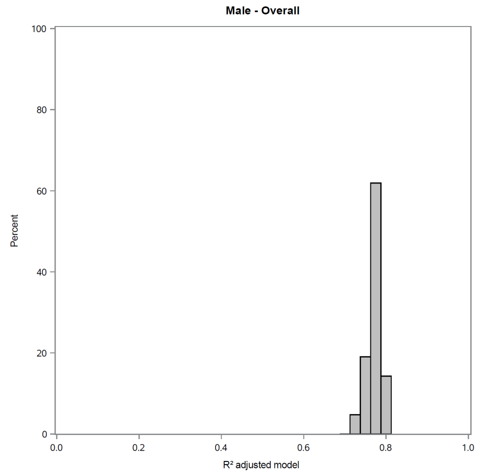

R^2 obtained in the leave-one-out models, range: 0.72-0.81
Leave-one-out cross-validated R^2 = 0.71

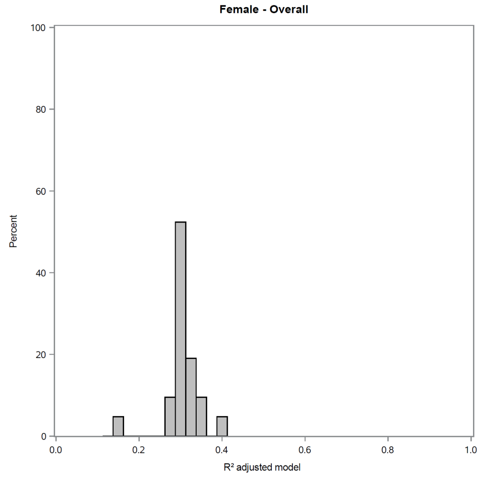

R^2 obtained in the leave-one-out models, range: 0.15-0.40
Leave-one-out cross-validated R^2 = 0.26

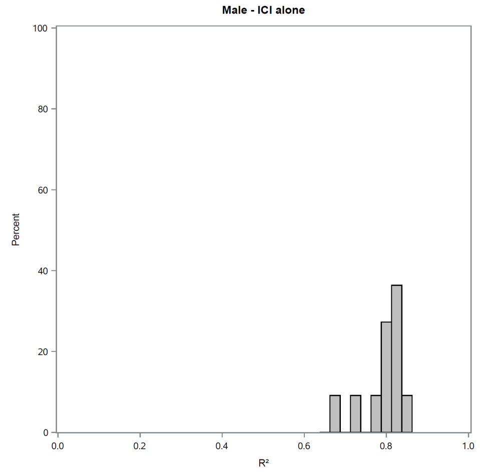

R^2 obtained in the leave-one-out models, range: 0.67-0.84
Leave-one-out cross-validated R^2 = 0.72

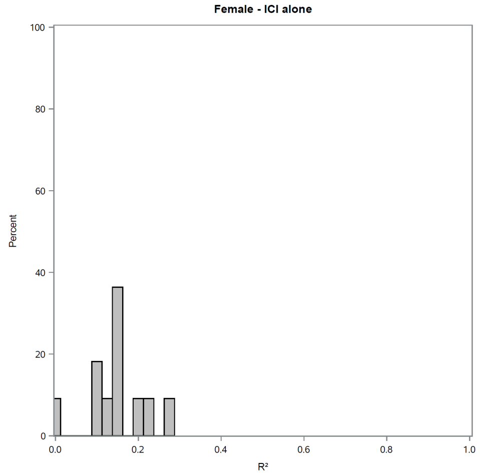

R^2 obtained in the leave-one-out models, range: 0.00-0.27
Leave-one-out cross-validated R^2 = 0.24

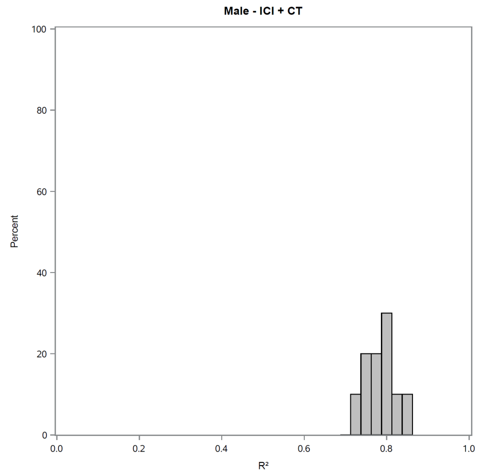

R^2 obtained in the leave-one-out models, range: 0.72-0.84
Leave-one-out cross-validated R^2 = 0.67

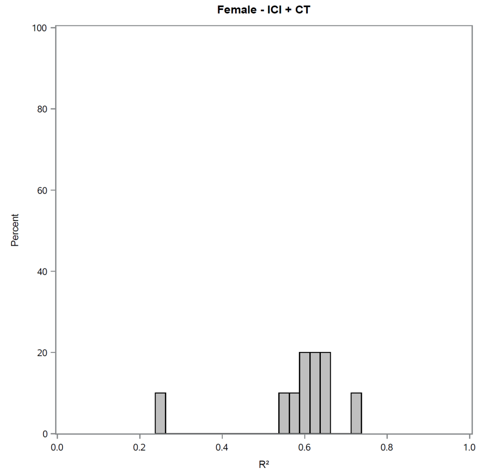

R^2 obtained in the leave-one-out models, range: 0.26-0.73
Leave-one-out cross-validated R^2 = 0.44

Abbreviations: CT, chemotherapy; ICI, immune checkpoint inhibitors.

For female and male – both overall and by type of treatment administered in the experimental arm – we reported histograms of the R^2 values obtained across the leave-one-out models. The range of R² values illustrates the variability in model fit across leave-one-out

iterations. The cross-validated R^2 are calculated as the squared correlation between observed and predicted OS treatment effects across comparisons.
